# Annotations

**Published:** 1907-03-16

**Authors:** 


### ANNOTATIONS.

### "Quiet" Intracranial Tumours.

The diagnosis of intracranial tumour has so formidable a sound, and is so often associated with urgent and alarming symptoms, that there is danger of forgetting that there are not infrequent cases in which an entirely opposite state of matters exists. Especially is this to be noted in children. Every now and again at one of the medical societies a patient is shown in whom, though double optic neuritis is present, there has been only a relatively slight complaint of headache, and perhaps little or no vomiting. Now, though double optic neuritis may doubtless exist apart from intracranial tumour, its existence, unless otherwise explained, most strongly suggests that diagnosis. Further, in the cases to which reference is now made there is usually some degree of headache and vomiting, and sometimes also some such significant event as the paralysis of one or more cranial nerves. But—and this is the point of importance—the headache and vomiting are often comparatively slight, and are interrupted by periods of what appears to be good health. The child perhaps has headache in the early morning, yet, after an hour or two, is able to go to school and to take part in games with his fellows. In such circumstances many parents, especially among the poorer classes, readily believe there is not much the matter, and, even if they consult a medical practitioner, are apt to minimise rather than to exaggerate the symptoms. It is here, then, the importance of ophthalmoscopic examination comes to the front, and its neglect is almost certain to lead to a profound error in diagnosis, prognosis, and treatment. The temptation to such neglect is provided by the lack of any complaint regarding the sight, but optic neuritis is quite consistent with good vision. Hence it should be remembered that the subjective disturbances produced by an intracranial tumour in a child may be very slight, and that the secret of successful diagnosis lies in the use of the ophthalmoscope.

### The London County Council and Cerebro-spinal Fever.

On more than one occasion in these columns we have urged that the epidemic displays of cerebro-spinal fever reported from Glasgow, Belfast, and other towns, demanded that the disease should be subjected to compulsory notification. It is therefore with much satisfaction we notice that the London County Council has taken action in this direction, and that, as a result of this, cerebro-spinal fever in the Metropolitan area must be notified in the same way as other epidemic diseases. Such a step cannot fail to excite the attention of the profession to the disease in question, and to bring cases of it, when they occur, to the notice of those charged with the care of the public health. Evidence is not wanting that the infection has already spread to the Metropolis, and a few individual examples of it have been seen in the out-patient departments of the children's hospitals. The uncertainty which overhangs the method of its spread from person to person, and the possibilities of epidemic extension which recent experiences have demonstrated, make it extremely desirable in the interests of the community that the disease shall be investigated in the most thorough and scientific fashion. This can hardly be accomplished unless all the facts come under the review of the public health authorities—an end which will now be attained through the machinery of compulsory notification. In a memorandum accompanying the official intimation from the County Council attention is drawn to the fact that in the milder and anomalous examples of the disease its identification is not easy; also to the occurrence of the "fulminant" variety, in which death ensues rapidly. For both of these deviations from the typical clinical picture of the disease it is necessary for the practitioner to be on the look-out. The recognition of the specific micro-organism in the cerebro-spinal fluid withdrawn by lumbar puncture is, of course, of the first moment, whilst Kernig's sign and a marked and persistent *tache cérébrale* are of great importance.

### Operations in the Out-patient Room.

The death of a patient while under an anæsthetic at the Central Throat and Ear Hospital, shows how unforeseen dangers may dwell in operating on a patient who has not been under strict and continuous surveillance for some time previously. No one but the victim himself was to blame for his death. He had come to the hospital with an enlarged tonsil, and had been instructed to return to undergo the operation needed for his relief. On his previous visit he had been warned, both orally and by printed instructions that had been given to him, that he must abstain from food on the day of the operation. He had been told that he must not take even milk or bread, though a cup of soup or beef-tea might be permitted. Had he obeyed these instructions all might have been well. As it was, soon after nitrous oxide gas had been administered and the operation begun, the patient's breathing failed, and although for over an hour every effort was made to restore him, he died without regaining consciousness. When a post-mortem examination was made it was found that the stomach contained nearly two pounds of undigested figs, which apparently the patient had eaten shortly before coming to the hospital. This sufficiently explained the fatal result. Clearly neither the operator nor the anæsthetist, who has administered anæsthetics in 18,000 cases without previous misadventure, can be blamed for what was due to the man's own folly. It is impossible to receive as in-patients all who come to a hospital for minor operations. To do so would be to limit the usefulness of the institution, and more serious cases might be excluded to make room for such as this, where all that was needed was that the patients should obey orders. The utmost that can be done, in addition to the printed and oral instructions now given, is to ask the patient when and what he has last eaten, and to decide from the answer whether to proceed or not. If a patient does not tell the truth about the matter, he alone can in such a case be blamed for any mischance that may result from the anæsthetic.

